# Effect of *L*-cysteine, *Boswellia serrata*, and Whey Protein on the Antioxidant and Physicochemical Properties of Pork Patties

**DOI:** 10.3390/foods9080993

**Published:** 2020-07-24

**Authors:** Fengqi Yang, Won-Young Cho, Han Geuk Seo, Byong-Tae Jeon, Ji-Han Kim, Yuan H. Brad Kim, Yanmei Wang, Chi-Ho Lee

**Affiliations:** 1 Department of Food Science and Biotechnology of Animal Resources, Konkuk University, Seoul 05029, Korea; yfq426@naver.com (F.Y.); ready1838@naver.com (W.-Y.C.); hgseo@konkuk.ac.kr (H.G.S.); Jihan.Kim@agresearch.co.nz (J.-H.K.); 2 HASUNG Co., Ltd. 201-164, Songi-ro, Songpa-gu, Seoul 05832, Korea; jbt@kku.ac.kr; 3 Meat Science and Muscle Biology Laboratory, Department of Animal Sciences, Purdue University, West Lafayette, IN 47907, USA; bradkim@purdue.edu; 4 Jilin Sino-ROK Institute of Animal Science, Changchun 130600, China; wangym@cstu.edu.cn

**Keywords:** *L*-cysteine, *Boswellia serrata*, whey protein, antioxidant potential, meat quality, pork patties

## Abstract

This study investigated the effects of *L*-cysteine (C) combined with *Boswellia serrata* (B) and whey protein (W) on the antioxidant and physicochemical properties of pork patties. Proximate composition, water holding capacity (WHC), pH, texture profile analysis, sensory evaluation, thiobarbituric acid-reactive substances (TBARS), DPPH radical-scavenging activity, volatile basic nitrogen (VBN), and color stability were assessed. Patty VBN gradually increased throughout the storage period. However, VBN for the C treatment increased relatively slowly, indicating that cysteine can delay spoilage and extend the shelf life of patties. The protein content of the whey powder treatment group increased to a greater extent than that of the C and control (CON) groups. Pork patties supplemented with antioxidants showed significantly higher WHC and significantly lower cooking loss and hardness than the CON. Moreover, the addition of 2% whey, 1% *B. serrata*, and 0.25% cysteine (WBC) significantly enhanced the relative DPPH radical-scavenging activity and sensory characteristics of the patties. After 7-day storage, the MetMb and TBARS values of all treatments were significantly lower than those of the untreated. The results indicated that there was synergy among the cysteine, *B. serrata*, and whey protein. This finding is of great importance to the production of high-quality pork patties with enhanced shelf life.

## 1. Introduction

Taste, flavor, appearance, quality, and shelf life are important in food choice and acceptance [1]. Ground meat products, such as patties, burgers, and meatballs, are comparatively more susceptible to oxidative degradation because mechanical operations, such as grinding, destroy cells and free radical-scavenging systems and increase product contact with air [2,3]. The National Research Council (1988) cites lipid oxidation as a major quality issue for meat. Processed meat products must be protected from oxidation during preparation, storage, and distribution in order to attain the expected shelf life. Controlled lipid oxidation may produce desirable product flavors, but lipid peroxidation lowers edible meat quality and creates unpleasant flavors. It also causes protein oxidation, alters and cross-links amino acid side chains, and influences protein emulsification, gelation, and water retention capacity [4,5,6].

Increasing consumer-driven demand for healthy food has led to the formulation of synthetic antioxidants. However, these may have adverse health effects on primates, such as liver, spleen, and lung injury. Consequently, research on natural antioxidants was initiated [7,8]. Natural and renewable resources are of considerable interest at this time. Hence, natural antioxidants are of great importance in food processing. They may improve food safety without compromising nutritive value or sensory quality [9]. Research and development have been conducted on natural antioxidants, such as pine bark, grape seed, rosemary, olive leaf, and oregano, for use in meat products [10]. Avocado by-products, blackberry, hawthorn, and dog rose have been investigated to limit oxidation in pork patties [11,12].

Amino acids may enhance animal growth performance as well as meat quality, flavor, and color. They may also increase animal myofibrillar protein solubility. For these reasons, they have received much attention in the meat industry [13,14]. Cysteine is a reducing agent [15]. It is sulfur-containing amino acid with a thiol group and metal chelation activity [16]. In addition, it has antioxidant activity in muscle cell cytoplasms and free radical-scavenging ability. It is also a meat flavoring precursor [17,18]. Anorexia, low bioavailability, and disease status significantly increase the demand for sulfur-containing amino acids. Low dietary cysteine intake may retard juvenile growth and cause liver damage, loss of muscle function, and other disorders [19,20]. One way to increase dietary cysteine intake is to add it to meat patties. Cysteine also inhibits the formation of heterocyclic amines and mutagenic agents in meat processing [21].

Whey contains β-lactoglobulin and α-lactalbumin, which are considered good antioxidants. It also has the sulfur-containing amino acids methionine and cysteine and these constitute part of the antioxidant defense mechanism of proteins [6,17]. *Boswellia serrata* or Indian frankincense is a tree resin. It is used to treat arthritis and has anti-inflammatory, anticancer, and bactericidal properties [22,23]; however, it is seldom used in the food industry. The main biologically active components of *B. serrata* include essential oils, terpenes, boswellic acid, and phenolic compounds [24]. Essential oil and terpenes may scavenge free radicals, chelate metals, and decompose peroxides [24,25].

Though several previous studies have investigated whey supplementation in meat products, few reports have explored the addition of *B. serrata* or cysteine to processed meats. The aim of this study was to determine whether the meat products containing cysteine, *B. serrata*, and whey powder have a synergistic antioxidant effect. To this end, we evaluated the TBV, VBN, DPPH, physicochemical properties, and sensory values of the meat products. It was postulated that compound antioxidants reinforce oxidative stability and enhance the quality of pork patties.

## 2. Materials and Methods

### 2.1. Pork Patty Formulation and Processing

*Boswellia serrata* powder, *L*-cysteine powder (food grade), whey powder, and refined salt were purchased from a local market, Source Naturals (Torrance, CA, USA), and ESfood (Gyeonggi-do, Korea), respectively. The *Boswellia serrata* contained 35.65 mg/kg sulfur compounds according to the National Instrumentation Center for Environmental Management, Seoul, Korea. Fresh pork fillet and pork back fat were purchased from a local meat market in Seoul, Korea. Excess fat and lean tissues were manually separated, and the connective tissue was discarded. The fat and lean pork were ground separately in a meat grinder fitted with a 5-mm plate. Five batches of twenty patties each were prepared by adding salt, cysteine, *B. serrata*, and whey powder to the meat according to the formulations listed in Table 1. Combined with previously published paper [26], it was reported that the addition of *B. serrata* is set at 1% since the high concentration group has better water holding capacity and antioxidant effect. The level of cysteine was based on Majcher and Jelen [27], who stated that the addition of 0.25% result in the form of a pleasant aroma compared to other concentrations. The samples were then mixed thoroughly for 12 min with a handheld mixer (SM 246; Poking Industrial Co., Ltd., Hong Kong, China). Both sides of the pork mixture (100 ± 1 g) were covered by a layer of polyethylene film and formed with a patty maker (120 mm × 80 mm × 25 mm; Spikomat Ltd., Nottingham, UK). Five groups of patties were prepared, vacuum-packed with polyethylene/nylon vacuum bags (Cryovac, Duncan, SC, USA) stored at 4 °C for 28 days, and analyzed at 7-day intervals (0 days, 7 days, 14 days, 21 days, and 28 days).

### 2.2. Proximate Composition

The proximate composition of the pork patties was determined according to AOAC methods (2012) [28]. The moisture content was evaluated by heating the patties to 105 °C in a drying oven and calculating the weight loss. The ash content was determined by drying and heating. The protein content was evaluated with a Kjeldahl nitrogen analyzer. The fat content was analyzed by the Soxhlet method.

### 2.3. pH

Two grams of pork patty per treatment was mixed with 18 mL distilled water in a homogenizer (AM-1, Nihon Seiki Kaisha Co., Ltd., Nagoya, Japan) at 3220 *g* for 60 s. The pH of each sample was measured with a pH meter (LAQUA F-71; Horiba Co., Kyoto, Japan).

### 2.4. Color

Meat color was evaluated with a colorimeter (Minolta Chroma Meter CR-210; Konica Minolta Inc., Tokyo, Japan), calibrated against a white plate (CIE L* = +97.83; CIE a* = −0.43; CIE b* = +1.98). CIE L* (lightness), CIE a* (redness), and CIE b* (yellowness) were measured for the surfaces of samples stored for 0 days, 7 days, 14 days, 21 days, and 28 days.

### 2.5. Water-Holding Capacity (WHC)

The Water-Holding Capacity (WHC) of the raw pork patty was determined according to the method of Akwetey and Yamoah [29]. Five-gram samples were weighed out, placed in a tube containing 10 mL distilled water, and mixed thoroughly. The suspension was centrifuged at 2000 rpm and 15 °C for 15 min in a refrigerated centrifuge (Sorvall RC-3; Thermo Fisher Scientific, Waltham, MA, USA). The supernatant was then decanted carefully, and the residue was weighed. WHC was expressed as a percentage and calculated as follows:

(1) $$\mathrm{WHC}\;(\%)=\frac{\mathrm{weight}\;\mathrm{of}\;\mathrm{sample}\;\mathrm{after}\;\mathrm{removing}\;\mathrm{supernatant}}{\mathrm{weight}\;\mathrm{of}\;\mathrm{sample}\;\mathrm{mixed}\;\mathrm{with}\;\mathrm{distilled}\;\mathrm{water}}\times 100$$

### 2.6. Cooking Loss and Texture Profile Analysis (TPA)

Patties were cooked on one side for 5 min and the other side for 7 min. They were then flipped and cooked for another 3 min in an electric oven (HM-602; Zhongshan Guanglong Gas & Electrical Appliances Co. Ltd., Guangdong, China). The cooked patties were then left to cool to room temperature (22–25 °C) for weighing and texture profile analysis. Cooking weight loss was calculated as follows:

(2) $$\mathrm{Cooking}\;\mathrm{loss}\;(\%)=\;\frac{\mathrm{patty}\;\mathrm{weight}\;\mathrm{before}\;\mathrm{cooking}-\mathrm{patty}\;\mathrm{weight}\;\mathrm{after}\;\mathrm{cooking}}{\mathrm{patty}\;\mathrm{weight}\;\mathrm{before}\;\mathrm{cooking}}\times 100$$

Triplicate samples 20 mm × 20 mm × 10 mm in size were cut from each cooked patty and subjected to TPA in a texture analyzer (CT3-1000; Brookfield Engineering Laboratories, Inc., Middleboro, MA, USA) fitted with a 5-kg loading cell. The samples were compressed twice at a pre-test speed of 5.0 mm s^−1^, a post-test speed of 2.0 mm s^−1^, a test speed of 2.0 mm s^−1^, and a force of 5.0. The parameters of the texture profile analysis included springiness, cohesiveness, gumminess, chewiness, and hardness and were calculated according to the method of Bourne [30].

### 2.7. Sensory Evaluation

Sensory properties (color, flavor, tenderness, juiciness, taste, and overall acceptability) of the pork patties were evaluated by a panel of 18 trained technicians (eight males and ten females; average age = 27.94 y) from the Department of Food Science and Biotechnology of Animal Resources, Konkuk University, Seoul, Korea. The cooked samples were cooled to room temperature, cut into blocks, randomized, and served to each panelist. The panelists evaluated the samples randomly and after rating each sample, rinsed their mouths with water and waited 1–2 min before evaluating the next sample. A nine-hedonic scale was used to assess each sensory parameter (color (1 = undesirable to 9 = desirable); flavor (1 = undesirable to 9 = desirable); tenderness (1 = tough to 9 = tender); juiciness (1 = dry to 9 = juicy); taste (1 = undesirable to 9 = desirable); overall acceptability (1 = undesirable, 9 = desirable)).

### 2.8. Thiobarbituric Acid-Reactive Substances (TBARS)

The TBARS assay determines secondary lipid oxidation. Thiobarbituric acid (TBA) reacts with the lipid oxidation product malondialdehyde (MDA) and forms a red chromophore that is measured spectrophotometrically. The UV/Vis spectrophotometer (Mecasys, Daejeon, Korea) was calibrated with a 1,1,3,3-tetraethoxypropane standard curve as described by Buege and Aust [31]. A 5-g meat sample was homogenized with 15 mL distilled water and 100 μL BHT at 13,000 rpm for 1 min. Then, 2 mL of the homogenate was reacted with 4 mL trichloroacetic acid/2-thiobarbituric acid reagent in an 80 °C water bath for 15 min. The samples were then cooled in cold water, centrifuged at 2000× *g* and 25 °C for 10 min, and filtered through Whatman No. 4 filter paper. Optical densities were measured at 531 nm in the aforementioned spectrophotometer.

### 2.9. Volatile Basic Nitrogen (VBN)

The VBN content was determined using Conway’s microdiffusion method with slight modifications [32]. Vaseline was applied to the edge of the outer ring. Five-gram patties were homogenized at 10,000 rpm for 1 min with 15 mL distilled water and filtered through Whatman No. 1 paper to remove fat and tissues. The sample solution and Conway reagent were then pipetted into the unit, covered, and fixed with a clip. The Conway tool was agitated, and the suspension was incubated at 37 °C for 120 min and titrated with 0.02 N sulfuric acid.

### 2.10. 2-Diphenyl-1-Picrylhydrazyl (DPPH) Radical-Scavenging Activity

DPPH radical-scavenging activity was assayed according to the method of Overland et al. [33]. Briefly, each 5-g sample was mixed with 20 mL methanol, homogenized at 13,000 rpm for 1 min, and sonicated (3210R-DTH; Branson Ultrasonics CO., Danbury, USA) for 10 min. The sonicated mixture was then centrifuged at 13,000 *g* and 4 °C for 10 min and the sediment was extracted with another 20 mL methanol. The supernatants were pooled in a test tube and diluted with methanol to 50 mL. Then, 0.1 mL of the methanolic extract was reacted with 2.4 mL methanolic DPPH solution (25 mg L^−1^) in the dark at 25 °C for 2 h. Absorbance was measured at 517 nm in a spectrophotometer (Multiskan GO; Thermo Fisher Scientific, Waltham, MA, USA).

DPPH radical scavenging activity = [1 − (absorbance_sample_/absorbance_control_)] × 100 (3)

### 2.11. Metmyoglobin (MetMb)

The metmyoglobin content was determined by the method of Warriss [34]. Each 4-g sample was homogenized with 20 mL of cooled 0.04 M phosphate buffer (pH 6.8) at 13,000 rpm for 10 s. The homogenate was left to react at 4 °C for 1 h, centrifuged at 5000 *g* and 5 °C for 30 min, and passed through Whatman No. 1 filter paper. Absorbance was measured in a spectrophotometer (UV/Vis Spectrophotometer, Mecasys, Daejeon, Korea) at 525 nm, 572 nm, and 730 nm. Metmyoglobin content was obtained as follows:

Metmyoglobin (%) = [1.395 − (A_572_ − A_700_)/(A_525_ − A_700_)] × 100 (4)

### 2.12. Statistical Analysis

All five treatments (twenty patties/per treatment) were repeated in triplicate. The effects of cysteine, *B. serrata*, and whey powder were evaluated by one-way ANOVA in SPSS v. 24.0 (SPSS Inc., Chicago, IL, USA) and means and SEM were recorded. Significant differences between means were analyzed by Tukey’s test. *p* < 0.05 was the acceptance level.

## 3. Results and Discussion

### 3.1. Proximate Analysis

The proximate composition of the pork patties supplemented with cysteine, *B. serrata*, and whey powder is shown in Table 2. There were no significant differences among samples in terms of crude fat content. There were no significant differences among the W, WC, and WBC groups in terms of moisture, however, they were all significantly decreased relative to C and CON (*p* < 0.001). The addition of whey and *B. serrata* might have increased the solid content and decreased the meat and fat content accordingly. Similar findings were reported by Barber [7] who found that breadfruit flour supplementation reduced the relative meat moisture content. Water content reduction lowers water activity which, in turn, decreases microbial activity in the meat and extends shelf life. The crude fat and ash content in the patties treated with cysteine powder did not significantly differ from those of the control. Whey and *B. serrata* might be good mineral sources. Whey powder comprises various proteins that significantly increase the comparative ash (*p* < 0.01) and protein (*p* < 0.001) content in the W and WBC treatments. Ha et al. [35] reported that chicken breast injected with whey showed a higher protein content than the untreated control. 

### 3.2. WHC, Cooking Loss, and Texture Profile Analysis (TPA)

WHC, cooking loss, and TPA values for the patties are presented in Table 3. The WHC is a critical determinant of product yield and eating quality [36]. The WBC treatment presented with the lowest cooking loss and highest WHC. However, the opposite was true for the CON (*p* < 0.001). Moreover, there were no significant differences among the other treatments in terms of these parameters. Hence, cysteine, *B. serrata*, and whey improved the water-binding capacity and decreased the cooking loss of the pork patties. These results corroborate those reported by Vlahova-Vangelova et al. [37]. They found that the whey treatment significantly increased WHC compared to the control. *B. serrata* contains phenolic compounds that may interact with proteins and reduce their surface hydrophobicity [38]. Ning et al. [39] reported that amino acids improve meat water-holding capacity and texture. Cooking loss substantially influences meat quality. Therefore, the aforementioned supplements may help enhance meat product palatability and juiciness.

The texture profile analysis demonstrated the effects of cysteine, *B. serrata*, and whey on the textural properties of the patties. Table 3 shows that there were no remarkable differences among treatments in terms of cohesiveness. The C, WC, and WBC samples had significantly lower springiness, chewiness, gumminess, and hardness (*p* < 0.001) than the others. However, the differences between WC and WBC in terms of these parameters were not significant. Thus, it is mainly cysteine that affects pork patty texture. As a rule, amino acids increase the solubility of myofibrillar proteins in vertebrate skeletal muscle [14] as previously reported by Heywood et al. [40]. Relative to the control, beef patty combined with high-cysteine texturized soy protein presented with increased tenderness and decreases in cohesiveness. Moreover, the essential oil content in *B. serrata* may reduce meat hardness. Nieto et al. [6] demonstrated that the addition of oregano or rosemary essential oil markedly inhibited the formation of cross-linked myosin heavy chains.

Here, the pork patties treated with cysteine had the lowest hardness and chewiness and were too brittle. Compared with the control, the whey powder treated group significantly increased patty hardness, followed by other treatments (*p* < 0.001). Muscle myosin was the first target to undergo meat protein oxidation. The CON group showed a high degree of hardness, possibly because the myosin heavy chain (MHC) in meat is readily oxidized at high oxygen tensions. Consequently, the protein is cross-linked and the meat toughness or hardness increases [41,42]. Furthermore, cooked meatballs supplemented with whey protein presented with increased relative hardness and chewiness [43]. These results indicated that cysteine and *B. serrata* may interact with whey and neutralize its negative effects on meat texture.

### 3.3. Sensory Evaluation

The sensory panel assessments of the cooked pork patties are presented in Table 4. Incorporation of the various additives to the pork patties did not affect their color attributes. The addition of whey powder alone had no significant effect on the sensory properties of the pork patties compared with the control. However, the patties of the WBC treatment had higher sensory scores (flavor, juiciness, and overall acceptability) than those of the other groups. The relatively higher water-holding capacities of the treatments might account for the fact that the patties of these groups were significantly juicier than those of CON (*p* < 0.001). Cysteine is an aroma precursor of sulfur-containing compounds, such as 2-methyl-3-furanthiol, which imparts a strong meaty aroma and substantially contributes to meat flavor [44]. Moreover, Menis-Henrique et al. [45] found that butyric acid and cysteine also confer desirable physical and sensory characteristics to certain snack foods. Low pH is conducive to the formation of aromatic compounds, such as 2-methyl-3-furanthiol [44]. *B. serrata* contains boswellic acid, which may lower meat pH. Hence, a combination of cysteine and *B*. *serrata* could enhance overall patty flavor. Tenderness is another important sensory property that consumers consider when they select meat products. The observed tenderness measurements were consistent with those for hardness in the TPA. The C group had the highest tenderness score, while the W group had the lowest (*p* < 0.001). There were no significant differences among C, WC, and WBC in terms of taste, but C had the highest score for this factor (*p* < 0.001). As *B. serrata* has a bitter taste, it might have had a negative impact on this property. The foregoing results suggest that the combination of cysteine, *B. serrata*, and whey might synergistically affect the sensory characteristics of pork patty.

### 3.4. pH Analysis

**Figure 1:**
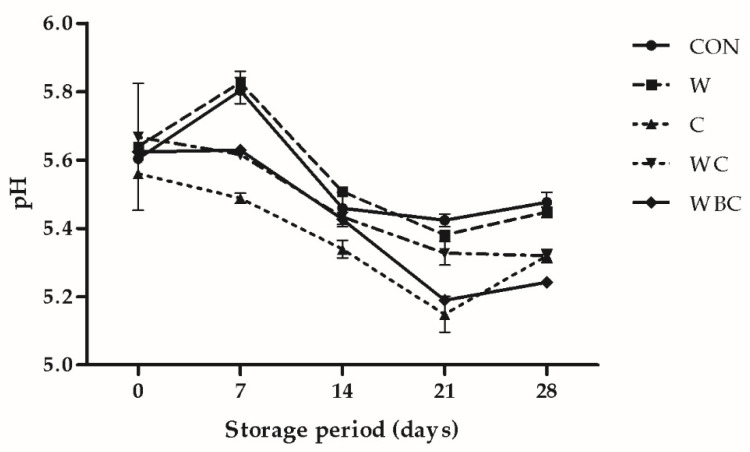
Relative changes in pork patty pH under various treatments and storage at 4 °C for 28 d. Error bars indicate SEM. CON (●), control (no additive(s)); W (■), 2% whey powder; C (▲), 0.25% cysteine powder; WC (▼), 2% whey powder plus 0.25% cysteine powder; WBC (◆), 2% whey powder, 1% *B. serrata* powder, and 0.25% cysteine powder.

The relative influences of the supplements on pork patty pH are shown in Figure 1. For all treatments, the pH tended to rise during 0–7-day storage and fall thereafter until day 21 when it slightly rose once again. A previous report showed a pH reduction with storage time in sausages treated with Moringa oleifera leaf. This supplement might be attributed to induced lactic acid accumulation [46]. In the later storage period (21–28 days), the increase in meat pH may be the reason for the bacteria and enzymes decomposing the protein and producing alkaline substances, such as biogenic amines. The meat pH did not significantly differ between the whey and control groups. However, the meat pH was significantly lower for the C and WBC samples than the other treatments during storage weeks 1–4. Thus, *B. serrata* and cysteine may have inhibited the growth of microorganisms to protect the meat. It is proposed that the observed reduction in pH was caused by both lactic acid formation and the boswellic acid from *B. serrata*. The foregoing results were consistent with those reported by Lara et al. [8] and by Park and Chin [47]. These authors stated that the pH of meats treated with natural antioxidants was significantly lower than that of the control. The active ingredient in the natural antioxidant was an acid; garlic extracts containing abundant sulfur compounds also decreased the pH values of patties than those of the control. Aktas et al. [48] demonstrated that low pH increases relative meat weight gain and improves product juiciness. This also, once again, verified the results shown previously, that treatments can obtain lower values of cooking loss, and high juiciness scores in sensory analysis.

### 3.5. Color Stability

**Figure 2:**
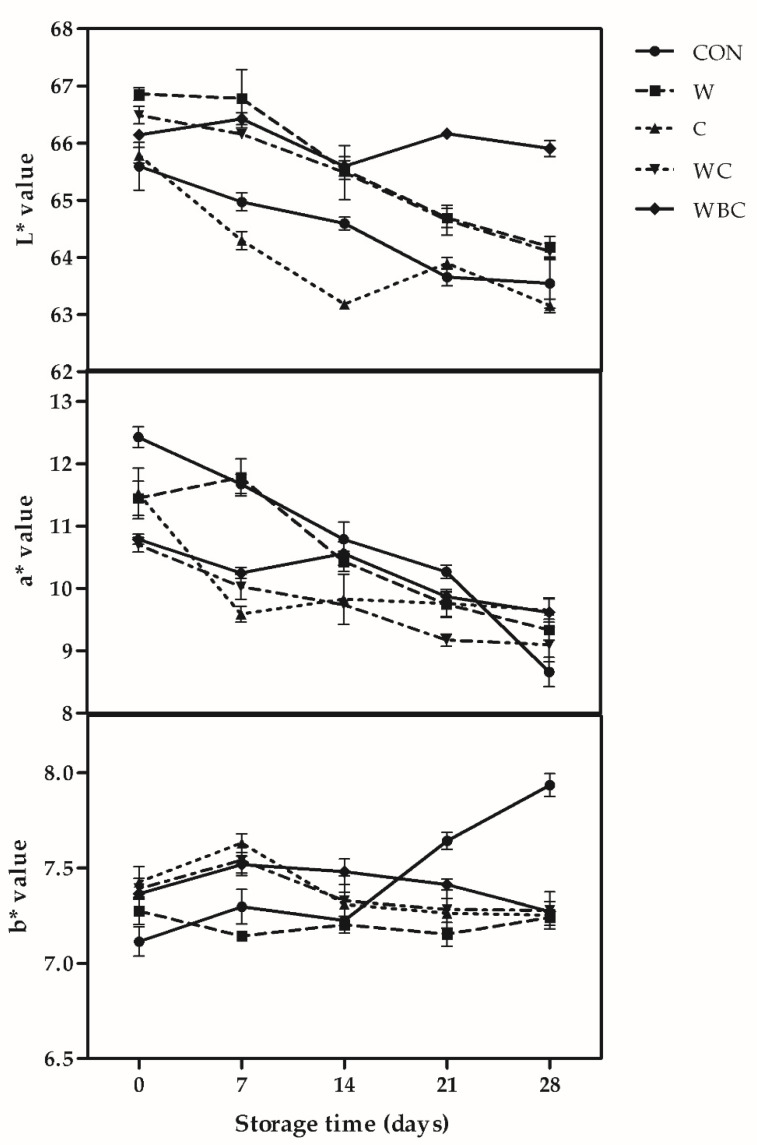
Relative changes in pork patty color stability during storage at 4 °C for 28 d. Error bars indicate SEM. CON (●), control (no additive(s)); W (■), 2% whey powder; C (▲), 0.25% cysteine powder; WC (▼), 2% whey powder plus 0.25% cysteine powder; WBC (◆), 2% whey powder, 1% *B. serrata* powder, and 0.25% cysteine powder.

Meat color is associated with lipid oxidation. Consumers tend to recognize bright red meat color as a sign of freshness. Therefore, meat product color must be stabilized [49]. Storage time and additive type affected the raw patty color parameters (Figure 2). During all storage, L* values showed an inconsistent trend. WBC was relatively stable, but a downward trend was shown in other treatments. On days 0–7, W had a higher L* than all other groups. Nevertheless, there were no differences among W, WC, and WBC in terms of L*. WBC presented with the highest L* after 14 d storage (*p* < 0.001). Uneven fat distribution might account for the observed fluctuations in L*. Meat color stability is also affected by myoglobin sensitivity to autoxidation [50]. The a* tended to decrease in all groups from days 7–28, however, a* declined slowly only in C, WC, and WBC. All treatments had stable a* and did not significantly differ from each other during the last week of storage. In contrast, the a* value for CON sharply dropped over time. Therefore, the results indicated that cysteine, *Boswellia*, and whey may have inhibited oxidative browning. Direct whey protein injection into chicken breast increased L* but decreased a*. Compared with other treatment whey protein concentrate group was the most effective at enhancing and maintaining meat redness [35,51]. There were no clear trends in b* for the W or WC treatments (7.15–7.28 and 7.28–7.54, respectively). There were only negligible changes in b* for C and WBC during the first and last weeks of storage, respectively, however, the b* for CON rose linearly over 14 days. The b* parameter indicates that patty yellowness is related to fat oxidation. Thus, the value of this factor may be reduced by improving meat oxidative stability [52]. The foregoing results show that natural antioxidants stabilize patty yellowness by preventing fat oxidation.

**Figure 3:**
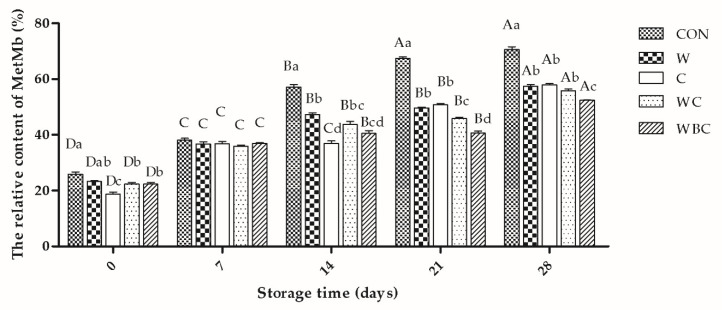
Effects of cysteine, *B. serrata*, and whey on MetMb content in pork patties during storage. Error bars indicate SEM. CON, control (no additive(s)); W, 2% whey powder; C, 0.25% cysteine powder; WC, 2% whey powder plus 0.25% cysteine powder; WBC, 2% whey powder, 1% *B. serrata* powder, and 0.25% cysteine powder. ^a–d^ Values with lowercase letters at same day of storage are significantly different from each other (*p* < 0.05), ^A–D^ Values with uppercase letters in same batch are significantly different from each other (*p* < 0.05).

Metmyoglobin is the product of oxymyoglobin oxidation during storage. Whereas oxymyoglobin is red, metmyoglobin is brownish. The formation of the latter may be positively correlated with lipid oxidation [53]. The MetMb content increased linearly with the storage period in all samples (Figure 3), possibly because of the oxidative attack of the myoglobin in the patties. This finding is consistent with that for the redness evaluation. However, the MetMb levels of the treatment groups were significantly lower (*p* < 0.001) than that of the control group at all storage time intervals, except day 7. Compared with the single additive group, the WBC group presented with a gradual increase in MetMb content from 22.28 to 52.44. The MetMb content was lowest during the 21–28-day storage period because of the synergistic interactions among cysteine, *B. serrata*, and whey. Myoglobin oxidation and lipid oxidation are interrelated, and the three natural antioxidants all contain sulfur compounds and have antioxidant effects, which may enhance the efficacy. The MetMb level in pork patties supplemented with 2.0% WPI hydrolysates was significantly lower than that of the control. Cysteine combination with NaNO_2_ effectively increased the a* and decreased MetMb in sausage relative to the control [39,54].

### 3.6. VBN Content

**Figure 4:**
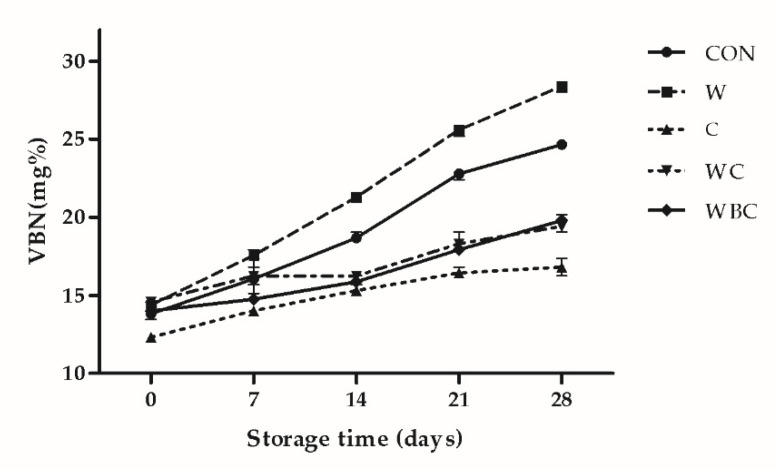
Relative changes in pork patty VBN among treatments during the storage period. Error bars indicate SEM. CON (●), control (no additive(s)); W (■), 2% whey powder; C (▲), 0.25% cysteine powder; WC (▼), 2% whey powder plus 0.25% cysteine powder; WBC (◆), 2% whey powder, 1% *B*. *serrata* powder, and 0.25% cysteine powder.

Figure 4 shows the relative changes in patty VBN during the storage period. For all treatments, VBN significantly increased relative to the control. The VBN of C was the lowest throughout the storage period. After 7 days, the VBN of W was the highest. The comparatively higher VBN content in W may be explained by the fact that the whey provided additional nitrogen. The VBN content is an indicator of spoilage. The microorganisms and enzymes in meat accelerate protein degradation and increase VBN [55]. The rate of increase in VBN did not differ between WC and WBC until the end of the storage period. After 7-day storage, however, the VBN for CON was significantly higher than those for C, WC, and WBC. It is known that certain sulfur-containing compounds are antimicrobial [56]. Whey powder contains cysteine and methionine, which are sulfur-containing amino acids, and sulfur compounds also detected in the *B. serrata*, so the three added ingredients may have a mutually promoting effect. Therefore, the addition of the antimicrobial cysteine and *B. serrata* might have delayed microbial protein degradation.

### 3.7. Antioxidant Activity

**Figure 5:**
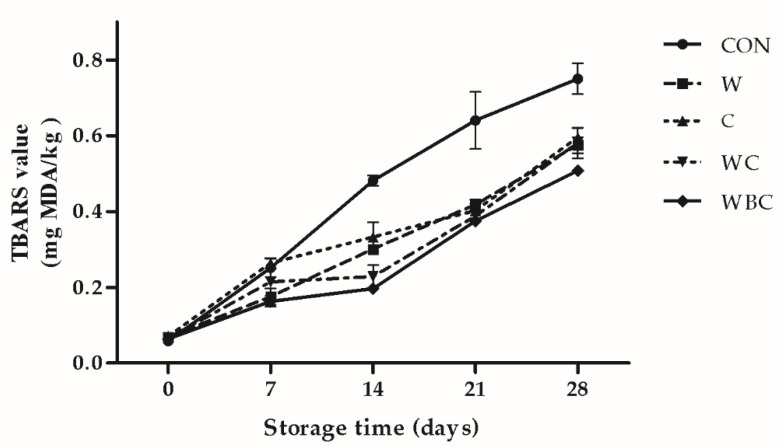
Relative changes in pork patty TBARS among various additive treatments during the storage period. Error bars indicate SEM. CON (●), control (no additive(s)); W (■), 2% whey powder; C (▲), 0.25% cysteine powder; WC (▼), 2% whey powder plus 0.25% cysteine powder; WBC (◆), 2% whey powder, 1% *B. serrata* powder, and 0.25% cysteine powder.

**Figure 6:**
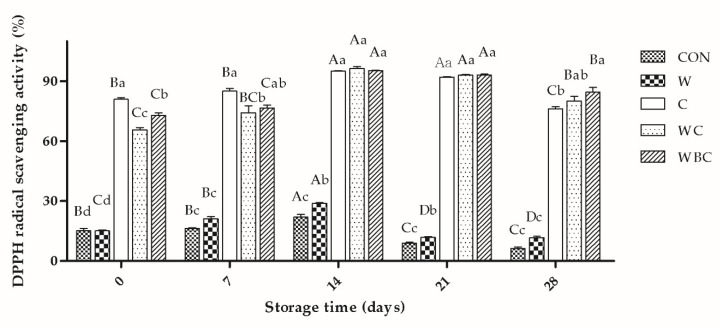
Effects of various treatments on DPPH radical-scavenging activity in pork patties during storage. Error bars indicate SEM. CON, control (no additive(s)); W, 2% whey powder; C, 0.25% cysteine powder; WC, 2% whey powder plus 0.25% cysteine powder; WBC, 2% whey powder, 1% *B. serrata* powder, and 0.25% cysteine powder. ^a–d^ Values with lowercase letters at same day of storage are significantly different from each other (*p* < 0.05), ^A–D^ Values with uppercase letters in same batch are significantly different from each other (*p* < 0.05).

The results of the antioxidant activity assay of pork patties subjected to the various treatments are shown in Figure 5 and Figure 6. At the onset of storage, there were no significant differences among groups in terms of TBARS. By day 7, the cysteine treatment presented with the highest TBARS (Figure 5). Ahn et al. [57] proposed that cysteine is initially pro-oxidant and antioxidant thereafter. During storage, however, the TBARS for all groups except WC increased linearly. The TBARS for WC slightly fluctuated during days 7–14. From days 14–28, the control presented with the highest TBARS and there were no significant differences between C and W or between WC and WBC in terms of TBARS. The lowest TBARS was measured for WBC. According to Ahn et al. [57], cysteine combined with tocopherol or ascorbate helped reduce meat patty TBARS. Here, the pork patties treated with a combination of cysteine, *B. serrata*, and whey were better protected against lipid oxidation than those treated with any of the individual ingredients. One possible explanation is that all three substances contain sulfur compounds. Antioxidants scavenge free radicals, prevent thiol oxidation, inhibit protein disulfide formation, and hinder protein crosslinking. This mechanism corresponds with the foregoing results finding that these treatments can reduce hardness.

DPPH is widely used to detect antioxidant activity as it is simple and highly sensitive [55]. The DPPH radical-scavenging activity in the pork patties first increased and then decreased with storage time under all treatments (Figure 6). The observed reductions in antioxidant may be explained by the fact that the antioxidants were hydrolyzed during storage in order to inhibit oxidative rancidity. Other authors reported that total phenolics in beef patties were consumed in storage and the interaction between milk protein and polyphenols reduced antioxidant activity in yogurt during refrigeration [58,59]. Meat may have endogenous (polyamines, peptides, uric acid) or exogenous hydrophilic (phenolics, ascorbate) antioxidants. Dipeptides in meat may be effective hydrophilic antioxidants [60] and might have accounted for the observed increase in DPPH of the CON group from days 0–14. From 14–21 days of storage, the DPPH of W was significantly higher than that of CON. It was suggested that methionine and cysteine, which are sulfur-containing amino acids are antioxidants and scavenge free radicals [6]. Ahmed et al. [61] demonstrated that whey-permeate treatments reduced DPPH significantly more than chlorine and water treatments because whey permeate has antioxidant activity. However, the DPPH reduction in the latter treatment was significantly lower than those for the other treatment groups. Here, the free radical-scavenging ability of cysteine was significantly higher than that of whey. The DPPH radical-scavenging activity was strongest for C during the first week of storage. Amino acid residues inhibit lipid oxidation by interacting with and scavenging lipid-derived free radicals [17]. Blois [62] introduced the DPPH method using cysteine as an antioxidant model and also showed that cysteine has good ability to scavenge free radicals. From day 21 of storage onwards, the DPPH radical-scavenging activity of WBC was the highest of all. Previous reports showed that plant extracts, such as drumstick leaves, pomegranate rind powder extract, and olive cake powder, can enhance DPPH radical-scavenging activity [58]. *B. serrata*, whey, and cysteine all have a DPPH radical-scavenging capacity and their combination has synergistic antioxidant efficacy, of which cysteine plays a major role.

## 4. Conclusions

Cysteine, *B. serrata*, and whey furnished antioxidant and free radical-scavenging capacity to pork patties during refrigerated storage. The addition of natural antioxidants to pork meat significantly improved the physicochemical and color properties and sensory characteristics compared to the untreated control. The combination of cysteine, *B. serrata*, and whey may synergistically inhibit pork patty fat oxidation, prevent discoloration, improve quality, enhance product value, and extend shelf life. Moreover, the hepatoprotective and antiarthritic efficacies of cysteine and *B. serrata* may help transform pork patties into healthful functional foods. Further study might be needed to validate this hypothesis through animal experimentation and compare with traditional antioxidants in the development of these novel products.

## Figures and Tables

**Table 1:** Formulations (wt %) of pork patties containing cysteine, *Boswellia serrata*, and whey powder.

Ingredient	Treatment ^1^				
	CON	W	C	WC	WBC
Pork fillet	75	75	75	75	75
Back fat	25	25	25	25	25
Salt	1	1	1	1	1
Cysteine	-	-	0.25	0.25	0.25
Whey	-	2	-	2	2
*B. serrata*	-	-	-	-	1
Total	101	103	101.25	103.25	104.25

^1^ CON, control (no additives); W, 2% whey powder; C, 0.25% cysteine powder; WC, 2% whey powder plus 0.25% cysteine powder; WBC, 2% whey powder, 1% *Bowsellia serrata* powder, and 0.25% cysteine powder.

**Table 2:** Proximate composition (%) of pork patties supplemented with cysteine, *Boswellia serrata*, and whey powder.

Treatment ^1^	Moisture	Fat	Protein	Ash
CON	54.27 ^a^	25.23	17.89 ^c^	1.82 ^b^
W	53.22 ^b^	24.70	19.03 ^a^	1.90 ^a^
C	54.45 ^a^	25.18	18.13 ^bc^	1.81 ^b^
WC	53.39 ^b^	24.54	18.47 ^b^	1.87 ^ab^
WBC	53.04 ^b^	24.15	18.50 ^ab^	1.90 ^a^
SEM ^2^	0.26	0.58	0.16	0.02
***p*-value**	***	0.365	***	**

^1^ CON, control (no additives); W, 2% whey powder; C, 0.25% cysteine powder; WC, 2% whey powder plus 0.25% cysteine powder; WBC, 2% whey powder, 1% *B. serrata* powder, and 0.25% cysteine powder. ^2^ SEM: standard error of the mean. ^a–c^ Means within the same column with different letters are significantly different from each other (*p* < 0.05). ** *p* < 0.01; *** *p* < 0.001.

**Table 3:** Effects of cysteine, *Boswellia serrata,* and whey on pork patty WHC (%), cooking loss (%), and texture profile.

Treatment ^1^	Springiness (mm)	Cohesiveness	Chewiness (kg)	Gumminess (kg)	Hardness (kg)	WHC^3^	Cooking loss
CON	0.72 ^a^	0.25	3.79 ^a^	4.98 ^a^	19.61 ^b^	45.04 ^c^	41.73 ^a^
W	0.68 ^ab^	0.22	3.30 ^b^	4.60 ^b^	20.92 ^a^	47.60 ^b^	39.34 ^b^
C	0.59 ^d^	0.21	1.82 ^d^	3.16 ^d^	13.84 ^d^	47.58 ^b^	38.97 ^b^
WC	0.65 ^bc^	0.22	2.67 ^c^	4.15 ^c^	18.08 ^c^	48.61 ^ab^	38.17 ^bc^
WBC	0.63 ^cd^	0.23	2.59 ^c^	4.05 ^c^	17.99 ^c^	49.01 ^a^	37.60 ^c^
SEM ^2^	0.02	0.01	0.09	0.11	0.43	0.39	0.43
***p*-value**	***	0.078	***	***	***	***	***

^1^ CON, control (no additives); W, 2% whey powder; C, 0.25% cysteine powder; WC, 2% whey powder plus 0.25% cysteine powder; WBC, 2% whey powder, 1% *B. serrata* powder, and 0.25% cysteine powder. ^2^ SEM: standard error of the mean. ^a–d^ Means within the same column with different letters are significantly different from each other *** *p* < 0.001. ^3^ WHC: water-holding capacity.

**Table 4:** Sensory analysis of cooked pork patties supplemented with cysteine, *Boswellia serrata,* and whey.

Treatment ^1^	Color	Flavor	Tenderness	Juiciness	Taste	Overall Acceptability
CON	5.83	6.25	6.08	5.00	5.25	5.50
W	5.83	6.33	5.50	5.83	6.08	6.17
C	6.25	6.83	7.50	5.92	7.17	6.58
WC	6.17	6.92	6.75	6.25	7.42	6.75
WBC	6.08	7.25	7.25	6.42	6.83	7.08
SEM ^2^	0.30	0.29	0.33	0.24	0.34	0.30
***p*-value**	0.527	**	***	***	***	***

^1^ CON, control (no additives); W, 2% whey powder; C, 0.25% cysteine powder; WC, 2% whey powder plus 0.25% cysteine powder; WBC, 2% whey powder, 1% *B. serrata* powder, and 0.25% cysteine powder. ^2^ SEM: standard error of the mean. ^a–c^ Means within the same column with different letters are significantly different from each other (*p* < 0.05). ** *p* < 0.01; *** *p* < 0.001.
